# Optimizing the interpretation of *Clostridioides difficile* two-step diagnostic algorithm results through antimicrobial stewardship

**DOI:** 10.1017/ash.2022.350

**Published:** 2022-12-15

**Authors:** Christopher F. Lowe, Shayan Shakeraneh, Colin Lee, Azra Sharma, Victor Leung

**Affiliations:** 1 Pathology and Laboratory Medicine, Providence Health Care, Vancouver, British Columbia, Canada; 2 Pathology and Laboratory Medicine, University of British Columbia, Vancouver, British Columbia, Canada; 3 Infection Prevention and Control, Providence Health Care, Vancouver, British Columbia, Canada; 4 Antimicrobial Stewardship Program, Providence Health Care, Vancouver, British Columbia, Canada

## Abstract

Over a 4-year period, the antimicrobial stewardship team reviewed all positive (PCR+/Tox+) and indeterminate (PCR+/Tox−) cases with the most responsible physician for classification of patients as infection or colonization. Among 501 indeterminate samples, 213 (43%) were considered to be clinical infection, suggesting the need for ongoing clinical assessment of indeterminates.

Surveillance for *Clostridioides difficile* is foundational in the prevention of healthcare-associated transmission. The minimum recommendation for healthcare facilities is to conduct a surveillance program for healthcare-associated *C. difficile* infection (HA-CDI) to identify increasing rates or outbreaks.^1^ Formally, reporting of *C. difficile* rates is primarily based on laboratory results,^2^ which may contribute to interpretive challenges depending on the method of laboratory detection (ie, molecular, molecular followed by enzyme immunoassay [EIA] (GDH/toxin A/B), or EIA followed by molecular) particularly for indeterminate (PCR+/Tox−) results. Furthermore, using laboratory-based surveillance only can systematically increase the incidence of HA-CDI.

Our laboratory transitioned to a 2-step test reporting algorithm in 2018 (ie, molecular followed by EIA). The change in reporting also prompted an ‘enhanced’ CDI surveillance initiative implemented by the combined infection prevention and control (IPC) and antimicrobial stewardship program (ASP) teams. In addition to the recommended infection control precautions for *C. difficile*,^1^ the IPC team alerted the ASP team of all inpatient cases with a positive or indeterminate *C. difficile* result, prompting detailed case review to ascertain colonization or true infection. This process enabled more detailed *C. difficile* surveillance data to inform IPC practices while enabling the ASP team to optimize treatment for *C. difficile* infections (and reduce unnecessary concurrent antimicrobial use). We describe the results of our ‘enhanced’ *C. difficile* surveillance, in which we sought to assess the difference in *C. difficile* infection rates based on laboratory results compared to the addition of an ASP and clinical review.

## Methods

**Fig. 1.**
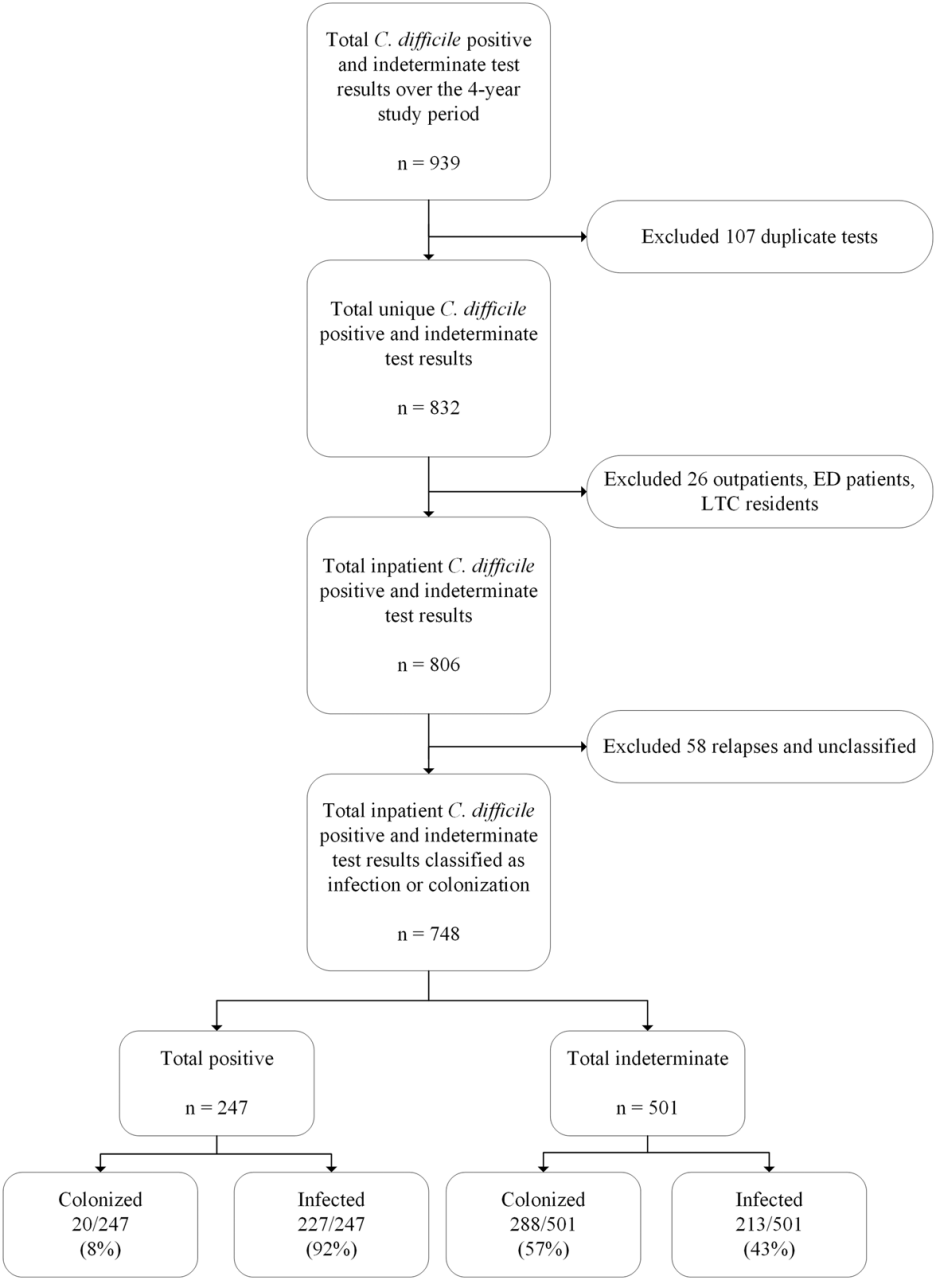
Overview of the *Clostridioides difficile* results included in the infection prevention and control (IPC) and antimicrobial stewardship (ASP) ‘enhanced’ surveillance.

We retrospectively reviewed the IPC CDI surveillance data (April 1, 2018, to March 31, 2022), excluding outpatient clinics, emergency department, and long-term care facilities. Duplicate samples, as well as relapsed cases, were excluded (Fig. 1). The 2-step algorithm included PCR (Xpert *C. difficile*, Cepheid, Sunnyvale, CA) for all appropriate stool specimens (ie, from patients with 3 or more loose or watery stools—Bristol types 6 and 7–above their baseline within a 24-hour period) followed by enzyme immunoassay (EIA) for toxins A/B and GDH (C. Diff Quik Chek Complete, Techlab, Blacksburg, VA) for all positive samples. The microbiology laboratory reviews all stool submissions and cancels orders for those with formed stool.

The process for clinical review has been previously described. For all positive (PCR+/Tox+) and indeterminate (PCR+/Tox−) inpatient cases, the IPC team alerts the ASP team who reviewed with the most responsible physician (MRP) to determine the patient’s clinical status (colonization or infection).^3^ The case was classified in the IPC surveillance database as colonization or infection when MRP and ASP had concordant assessments. If there was discordance regarding the clinical assessment between ASP and MRP, classification would be based on the final MRP decision. Assessment of infection was based on clinical symptoms (and stool charts), laboratory results (eg, leukocytosis), and an absence of an alternate diagnosis. During the study period, IPC practices and treatment recommendations for *C. difficile* were unchanged. Statistical analyses were performed using Microsoft Excel (Microsoft, Redmond, WA) and OpenEpi version 3.01 software.

## Results

During the 4-year study period, 748 inpatients were reviewed by the IPC and ASP teams, with 247 positive and 501 indeterminate results (Table 1). The proportion of positive and indeterminate cases was generally unchanged over time. On clinical review of all indeterminate cases, the proportion of patients colonized with *C. difficile* statistically significantly increased from 2018–2019 to 2021–2022 relative to those determined to have acute clinical infection (*P* < .05). Overall, 288 (57%) of 501 of all indeterminate results were considered colonization. With respect to patients with positive *C. difficile* results, only 20 (8%) of 247 positive cases were classified as colonization.

**Table 1.** *Clostridioides difficile* Cases Identified in the Microbiology Laboratory from 2018–2019 to 2021–2022 and Classification of Cases After Clinical Review (Colonized or Infected)

Fiscal Year	Total Cases (Positive and Indeterminate), No.	Positive (PCR+/Tox+), No.	Positive (PCR+/Tox+) and Colonized, No. (%)	Positive (PCR+/Tox+) and Infected, No. (%)	Indeterminate (PCR+/Tox−), No.	Indeterminate (PCR+/Tox−) and Colonized, No. (%)	Indeterminate (PCR+/Tox−) and Infected, No. (%)
2018–2019	182	53	7 (13)	46 (87)	129	70 (54)	59 (46)
2019–2020	167	50	3 (6)	47 (94)	117	62 (53)	55 (47)
2020–2021	187	70	6 (9)	64 (91)	117	70 (60)	47 (40)
2021–2022	212	74	4 (5)	70 (95)	138	86 (62)	52 (38)
Total	748	247	20 (8)	227 (92)	501	288 (57)	213 (43)

Note. PCR, polymerase chain reaction assay; PCR+/Tox+, positive; PCR+/Tox−, indeterminate.

## Discussion

Our institution’s ‘enhanced’ surveillance for *C. difficile* called for a detailed review of the clinical presentation for patients tested with a 2-step algorithm (ie, PCR and toxin EIA). There continues to be uncertainty regarding the clinical relevance of PCR+/Tox−, though *C. difficile* testing with molecular methods alone have been associated with overdiagnosis of infection.^4^ In our experience, interpretation of indeterminate (PCR+/Tox−) results requires a clinical assessment. We previously identified that 54% of PCR+/Tox− cases were considered to be true infections.^3^ Longitudinal follow-up in this study revealed that trends in infection versus colonization shifted over time, with more indeterminate cases representing colonization. Although the underlying reason for this shift is not clear yet and requires further investigation, familiarity over time with the 2-step algorithm and ASP review may have contributed to increasing comfort level among MRPs to classify cases as colonization. It further underscores the importance of diagnostic stewardship for *C. difficile* testing, and for the IPC and ASP teams to encourage clinicians to order testing only when there is clinical suspicion, rather than for any patient with a reported case of loose stool.

For patients with PCR+/Tox− results who were considered colonized, our earlier study suggested that there were no differences in adverse outcomes on follow-up 8 weeks after assessment, despite those patients not prescribed treatment for CDI. This finding is similar to a recent study assessing outcomes for untreated patients with PCR+/Tox−, which found noninferiority for unresolved diarrhea at 7 days and 30-day all-cause mortality.^5^ For facilities where the microbiology laboratory tests by a molecular method and for the presence of toxin production, indeterminate (PCR+/Tox−) results represent a significant potential for ASP to reduce inappropriate antibiotic use given that *C. difficile* therapy (eg, oral vancomycin) may have unintended sequelae due to its impact on the human intestinal microbiota.^6^ However, clinical review is essential to ensure that appropriate patient populations are targeted to avoid missing treatment given that 43% of indeterminate cases in this study were considered true infections.

Surveillance for HA-CDI is inherently impacted by laboratory methodology. Molecular methods alone may overestimate rates, while toxin EIA as a primary test may underestimate rates.^7^ Based on the CDC NHSN definitions, for laboratories that utilize a 2-step testing algorithm with a molecular assay first, PCR+/Tox− would not be a reportable case (though the exact same results, except with an EIA for GDH/toxin A/B as the primary test would be considered a reportable case).^2^ Our study has highlighted that although most PCR+/Tox− cases represent colonization, 43% of cases represent infection. Relying solely on the laboratory definition may lead to delayed treatment, underreporting of CDI, and potential for *C. difficile* transmission in the absence of precautions in this subset of PCR+/Tox− patients.

This study had several limitations. These results may not be generalizable to other settings; our results were based on 2 hospitals in our institution. In addition, the rates of colonization versus infection may be underestimated because final classification was adjudicated by the MRP, which has not yet been evaluated to determine the reliability or validity in comparison to established definitions for *C. difficile*. If the MRP decides to treat for CDI despite ASP indicating a likely case of colonization, the case would be classified as an infection, deferring to the clinical judgement of the physician caring for the patient.

Both IPC and ASP teams contribute to the prevention of *C. difficile* transmission in healthcare facilities.^1^ However, active antimicrobial stewardship interventions have been reported to be infrequent among the SHEA Research Network hospitals, which is a missed stewardship opportunity to reduce *C. difficile*–specific antibiotics as well as education regarding the need (or lack thereof) for concurrent antimicrobials.^10^ The collaboration enabled an ASP intervention for *C. difficile* treatment and allowed for more detailed surveillance data for HA-CDI—specifically, further clarification of CDI on a clinical review rather than solely on a laboratory result.

## References

[1] McDonald LC , Gerding DN , Johnson S , et al. Clinical practice guidelines for *Clostridium difficile* infection in adults and children: 2017 update by the Infectious Diseases Society of America (IDSA) and Society for Healthcare Epidemiology of America (SHEA). Clin Infect Dis 2018;66:e1–e48. 29462280 10.1093/cid/cix1085 PMC6018983

[2] Multidrug-resistant organism and Clostridioides difficile infection (MDRO/CDI) module. Centers for Disease Control and Prevention website. https://www.cdc.gov/nhsn/pdfs/pscmanual/12pscmdro_cdadcurrent.pdf. Published 2022. Accessed September 13, 2022.

[3] Zou J , Leung V , Champagne S , et al. Clinical heterogeneity of patients with stool samples testing PCR+/Tox− from a two-step *Clostridium difficile* diagnostic algorithm. Eur J Clin Microbiol Infect Dis 2018;37:2355–2359. 30238342 10.1007/s10096-018-3383-7

[4] Polage CR , Gyorke CE , Kennedy MA , et al. Overdiagnosis of *Clostridium difficile* infection in the molecular test era. JAMA Intern Med 2015;175:1792–1801. 26348734 10.1001/jamainternmed.2015.4114 PMC4948649

[5] Hogan CA , Hitchcock MM , Frost S , et al. Clinical outcomes of treated and untreated *C. difficile* PCR-positive/toxin-negative adult hospitalized patients: a quasi-experimental noninferiority study. J Clin Microbiol 2022;60:e0218721. 35611653 10.1128/jcm.02187-21 PMC9199396

[6] Isaac S , Scher JU , Djukovic A , et al. Short- and long-term effects of oral vancomycin on the human intestinal microbiota. J Antimicrob Chemother 2017;72:128–136. 27707993 10.1093/jac/dkw383 PMC5161046

[7] Fang FC , Polage CR , Wilcox MH. Point-counterpoint: what is the optimal approach for detection of *Clostridium difficile* infection? J Clin Microbiol 2017;55:670–680. 28077697 10.1128/JCM.02463-16 PMC5328433

[8] Infection prevention and control, Providence Health Care: annual report 2018–19. Providence Health Care website. https://www.providencehealthcare.org/sites/default/files/2018-19%20Annual%20Report.pdf. Published 2019. Accessed September 13, 2022.

[9] Warren BG , Turner NA , Addison R , et al. The impact of infection versus colonization on *Clostridioides difficile* environmental contamination in hospitalized patients with diarrhea. Open Forum Infect Dis 2022;9:ofac069. 35265730 10.1093/ofid/ofac069 PMC8900930

[10] Sullivan KV , Gallagher JC , Leekha S , et al. Use of diagnostic and antimicrobial stewardship practices to improve *Clostridioides difficile* testing among SHEA Research Network hospitals. Infect Control Hosp Epidemiol 2022;43:930–934. 34376271 10.1017/ice.2021.133

